# Impact of COVID19 Lockdown in Eating Disorders: A Multicenter Collaborative International Study

**DOI:** 10.1192/j.eurpsy.2022.976

**Published:** 2022-09-01

**Authors:** M. Etxandi, I. Baenas, L. Munguía, R. Granero, G. Mestre-Bach, I. Sánchez, S. Jimenez-Murcia, F. Fernandez-Aranda

**Affiliations:** 1 Bellvitge University Hospital, Department Of Psychiatry, L’Hospitalet de Llobregat, Spain; 2 Instituto de Salud Carlos III, Ciber Fisiopatología De La Obesidad Y Nutrición, Madrid, Spain; 3 Autonomous University of Barcelona, 3department Of Psychobiology And Methodology, Bracelona, Spain; 4 Universidad Internacional de La Rioja, Department Of Psychiatry, Logroño, Spain; 5 School of Medicine and Health Sciences, University of Barcelona, Department Of Clinical Sciences, Barcelona -Campus Bellvitge, Spain

**Keywords:** COVID Isolation Eating Scale (CIES), Eating Disorders, Covid-19, lockdown

## Abstract

**Introduction:**

COVID19 lockdown is having a significant impact on mental health, patients with eating disorders (ED) are particularly vulnerable.

**Objectives:**

1) To explore changes in eating and other psychological features due to confinement in patients with ED from various European and Asian countries; and 2) to assess differences related to diagnostic subtypes, age and geography.

**Methods:**

The sample comprised 829 participants, diagnosed with an ED according to DSM-5 criteria from specialized ED units in Europe and Asia. Participants were assessed using the COVID19 Isolation Scale (CIES).

**Results:**

On one hand, patients with Binge Eating Disorder experienced the highest impact on weight and ED symptoms due to confinement. Together with subjects diagnosed with Other Specified Feeding and Eating Disorders (OFSED), they also experienced a deterioration in general psychological state. On the other hand, there was less symptomatic impact on people with Bulimia Nervosa or Anorexia Nervosa and asian and younger individuals appeared to be more resilient in this situation.

**Conclusions:**

The impact of COVID varied by cultural context and individual variation in age and form of illness. Services may need to target preventive measures and adapting therapeutic approaches for the most vulnerable patients.

**Disclosure:**

No significant relationships.

